# Ventilator-associated pneumonia (VAP) and pleural empyema caused by multidrug-resistant *Acinetobacter baumannii* in HIV and COVID 19 infected patient: A case report

**DOI:** 10.1016/j.imj.2023.02.004

**Published:** 2023-03-04

**Authors:** Rosa Anna Passerotto, Francesco Lamanna, Damiano Farinacci, Alex Dusina, Simona Di Giambenedetto, Arturo Ciccullo, Alberto Borghetti

**Affiliations:** a Dipartimento di Sicurezza e Bioetica - Sezione di Malattie Infettive, Università Cattolica del Sacro Cuore, 00168 Rome, Italy; b Dipartimento di Scienze di Laboratorio e Infettivologiche, Fondazione Policlinico Universitario Agostino Gemelli IRCCS, 00168 Rome, Italy; c Infectious Diseases Unit, San Salvatore Hospital, 67100 L'Aquila, Italy

**Keywords:** *Acinetobacter baumannii*, colistin, cefiderocol, SARS-CoV-2, HIV

## Abstract

We analyzed the case of a 49-year-old woman with HIV infection off-therapy with poor viro-immunological compensation, not vaccinated for SARS-COV-2, hospitalized for lobar pneumonia and severe COVID19-related respiratory failure in intensive care unit (ICU). The hospitalization was complicated by bacteraemic ventilator-associated pneumonia (VAP) caused by multidrug-resistant *Acinetobacter baumannii* (MDR-AB) isolated on pleural fluid culture, treated with colistin and cefiderocol for about 3 weeks. The molecular research of MDR-AB on transtracheal aspirate was negative following this therapy.

The aim is to show the safety, efficacy and tolerability of colistin-based combination therapy with cefiderocol for *Acinetobacter baumannii* infection in HIV-infected patient.

## Graphical abstract

**Figure:**
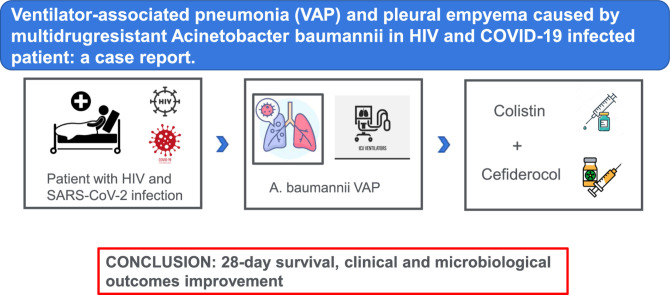

## 1 Introduction

*A. baumannii* survives for prolonged periods under a wide range of environmental conditions. The organism causes outbreaks of infection and health care-associated infections. Antimicrobial resistance greatly limits the therapeutic options, especially if isolates are resistant to the carbapenem class [1].

Colistin usually combined to other drugs such as tigecycline, ampicillin/sulbactam, meropenem or fosfomycin has been considered the backbone therapy despite its considerable nephrotoxic effect [2]. Recently, cefiderocol has been approved for the treatment of serious infections caused by carbapenem-resistant Gram-negative bacteria (CR-GNB) [3].

The burden of CRAB has been increasing during the COVID-19 pandemic; the weakening in antimicrobial resistance surveillance activities, the need for reorganizing ICU spaces and activities during pandemic waves and the overuse of antibiotics for COVID-19 pneumonia contribute to this increase [4,5].

Data on the efficacy, safety, tolerability of cefiderocol in patients with CRAB infections are still lacking. There are also no studies in this regard applied to patients with HIV infection.

The aim of this study is to evaluate the impact of cefiderocol-colistin regimen on the outcome of HIV and COVID-19 infected patient with CRAB infection.

## 2 Case description

49-year-old female patient, drug addict, smoker, with a 27-year history of HIV infection, voluntarily off-therapy for 25 years. Not vaccinated for SARS-CoV-2.

She was hospitalized on April 2022 for respiratory failure due to SARS-CoV-2-pneumonia combined to *Streptococcus pneumoniae*-related lobar pneumonia with bilateral interstitial involvement, areas of acute inflammatory hypodiaphania in the right mid-basal site and pleural effusion on the right at chest x-rays.

At the time of hospitalization HIV-RNA was 4,545,746 copies/mL, CD4 cell count was 25/mm^3^ and CD4-to-CD8 ratio was 0.19.

She started antibiotic therapy with ceftriaxone for *Streptococcus pneumoniae* for 7 days. About SARS-CoV-2-pneumonia steroid-therapy and anticoagulant-prophylaxis were performed. The antigenic nasopharyngeal swab for SARS-CoV-2 became negative after 3 weeks.

Regarding the management of respiratory failure, cycles of high flow oxygen therapy, non-invasive mechanical ventilation and pronation-supination were performed. Orotracheal intubation was executed due to worsening of respiratory exchanges. Eventually, due to difficulty in weaning from mechanical ventilation, percutaneous tracheostomy was prepared, and mechanical ventilation was subsequently disconnected. The patient was transferred to the ordinary ward with supplementation of low oxygen flows via tracheostomy, in spontaneous breathing.

After a sudden new worsening of clinic and respiratory exchanges, with need for high flow oxygen therapy via tracheostomy, the patient was transferred back to the intensive care unit. Following the detection at chest CT of an abundant amount of right pleural effusion, thoracic drainage was positioned, and samplings of the pleural fluid were taken. The microbiological analysis of the pleural fluid showed a positive culture test for multidrug-resistant *Acinetobacter baumannii*. The same microorganism was subsequently isolated also on rectal swab and blood cultures from peripheral venous blood. As for susceptibility testing (performed by broth microdilution, as for EUCAST guidelines), showing resistance of *Acinetobacter* to all antibiotic classes but polymyxins (see Table 1), antimicrobial therapy was set with colistin (the loading dose of 9 million units and then the maintenance dose with 5.5 million units every 12 hours, in light of the creatinine clearance > 90 mL/minute), with an initial worsening in renal function, and cefiderocol for 3 weeks.

**Table 1:** Acinetobacter baumannii antibiogram test on pleural fluid.

Antibiotics	EUCAST breakpoint (mg/L)	
	MIC	SIR
Amikacina	>16	R
Ciproflfloxacine	>1	R
Colistina	≤ 0.5	a
Gentamicina	>8	R
Meropenem	16	R
Trimeth-sulfamethoxazolo	>8	R

I, high probability of therapeutic success provided drug exposure is increased.

MIC, minimal inhibitory concentration; R, resistant; R, resistant; S, suscetible.

a Strain without acquired mechanisms of resistance to colistin.

**Fig. 1:**
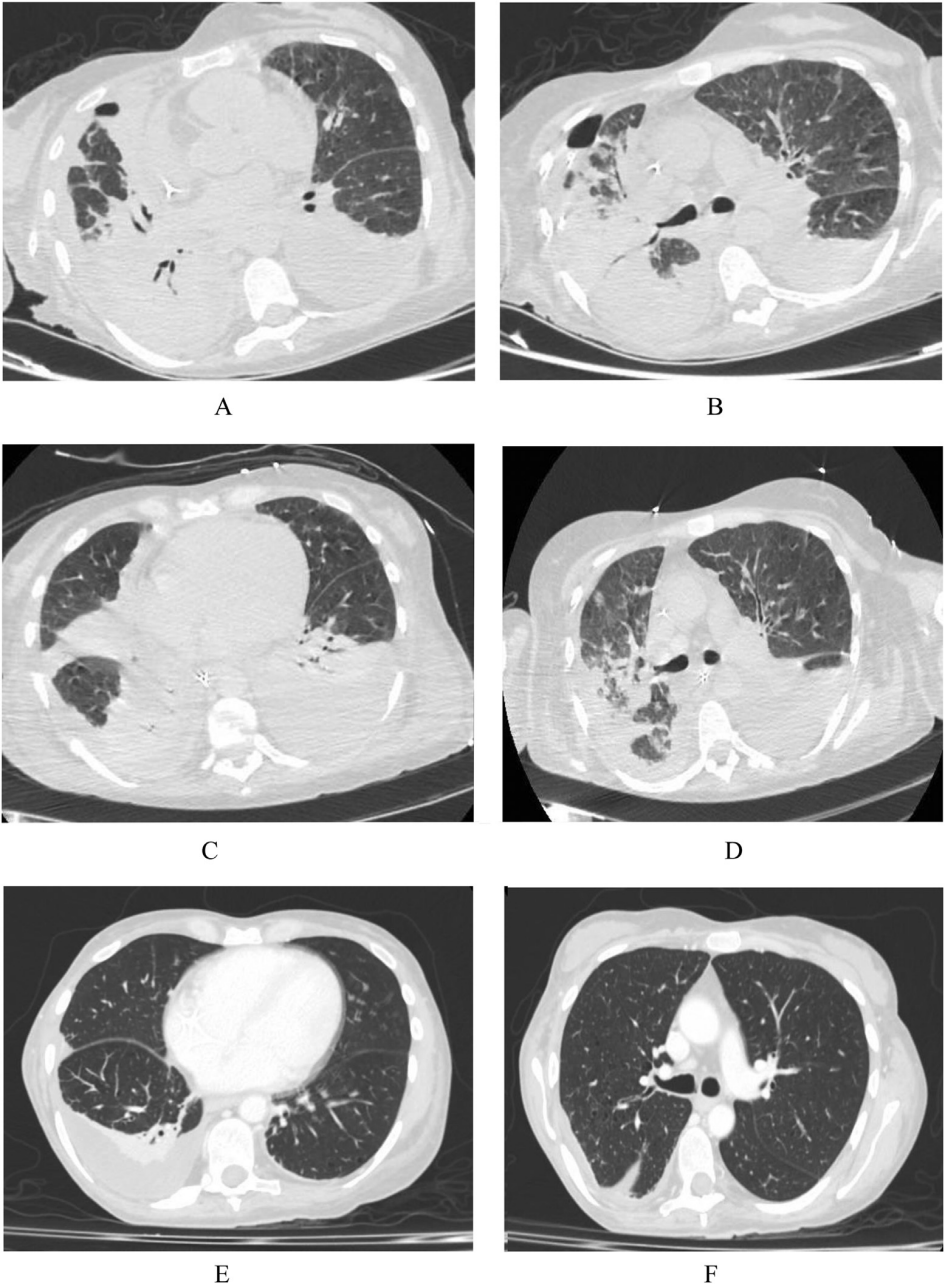
The patient's chest CT images. (**A-B)** (before treatment) show multiple bilateral areas of increased parenchymal density, partly "ground glass" and partly consolidative, with bilateral pleural effusion. (**C-D)** (under treatment). (**E-F)** (after treatment).

There was a progressive improvement in respiratory exchanges until reaching of spontaneous breathing with delivery of low-flow oxygen therapy and the chest drainage was removed. The patient was transferred to the ordinary infectious disease ward, where antibiotic therapy was continued. Molecular research for *Acinetobacter baumannii* on tracheal aspirate and bronchoalveolar lavage become negative after the therapy. The patient remained stably apyretic and an improvement in the laboratory indexes of inflammation was observed with a decrease in the C-reactive protein (CRP) and procalcitonin (PCT) values (before treatment CRP 143 mg/L and PCT 0.94 ng/mL; after treatment CRP 40.9 mg/L and PCT 0.63 ng/mL). Moreover a progressive radiological improvement was observed as shown in Figure 1.

During hospitalization a new viro-immunological determination showed HIV-RNA 2,096,183 copies/mL, CD4 90 cells/mm^3^ and CD4-to-CD8 ratio 0.10. After normalization of renal function, the patient resumed antiretroviral therapy using a tenofovir alafenamide fumarate (TAF)/emtricitabine (FTC) + dolutegravir (DTG) regimen.

## 3 Discussion

CRAB infection has been associated with an increased risk of mortality, particularly in COVID-19 patients with current infection or recent healing [6]. It is still unknown whether a history of HIV infection worsens outcome in patients with concurrent CRAB and COVID-19 infection.

According to the 2022 IDSA guidance on the treatment of multidrug resistant pathogens, cefiderocol use in the context of infections by *Acinetobacter baumannii* should be limited to cases where other options are not suitable. Similarly, ESCMID guidelines conditionally recommend against the use of cefiderocol, based on low-certainty evidence. In both Societies'recommendations, ampicillin-sulbactam is suggested as a preferred agent for mild-to-severe infections caused by CRAB, alone or as a part of combination therapy. However, in our Centre, susceptibility test for ampicillin/sulbactam is not available, even if evidence seems to suggest that high-dose ampicillin-sulbactam could still retain a non-negligible clinical efficacy.

Not all molecules display equivalent activity against *Acinetobacter baumannii*. Analyzing the epidemiology of CRAB in our hospital, almost all of the circulating *Acinetobacter baumannii* is a producer of OXA-23 which is not sensitive to therapy with ampicillin/sulbactam. For patients with CRAB resistant to sulbactam, ESCMID 2022 guidelines recommend against high-dose tigecycline. About the use of rifabutin or other rifamycins, ESCMID 2022 and IDSA 2021 guidelines do not recommend polymyxin-rifampin combination therapy. About fosfomycin, IDSA 2021 guidelines does not favor the use of fosfomycin as a component of CRAB therapy.

Among commercially available options, only colistin (if sensitive to antibiogram) and the recently approved cephalosporin, cefiderocol, have shown significant activity against this pathogen. Registration studies of cefiderocol have demonstrated efficacy in severe infections induced by *A. baumannii* although episodes of MIC increases, especially upon monotherapy administration, have been reported during therapy [7,8]. The SIDERO surveillance study [9] conducted on 236 strains (displaying non-susceptibility to carbapenems with MICs >8 mg/L) reported MIC ranges between 0.015 and >64 mg/L for cefiderocol and ≤0.25 to >8 mg/L for colistin. Susceptibility rates of 94.9% for cefiderocol and 93.6% for colistin were reported, whereas susceptibility rates above 90% were reported for OXA-23 and OXA-24 producers.

CRAB represents a true pathogen capable of contributing to excess mortality. It is challenging to determine if poor clinical outcomes are attributable to suboptimal antibiotic therapy or to underlying host factors such as immunocompromise in HIV infection. In this case report we use combination therapy with 2 agents but not among polymyxin plus fosfomycin or tigecycline or ampicillin/sulbactam or meropenem as ESCMID 2022 guidelines and 2022 IDSA guidance suggest. Moreover according to randomized clinical trial (RCT) CREDIBLE we get poor outcomes with cefiderocol as monotherapy in pneumonia and BSI. The 30-day survival of a patient with concomitant Acinetobacter baumannii, SARS-CoV-2 and HIV infection, and the microbiological clearance with colistin-cefiderocol combination therapy is a promising result, which adds new information on the clinical experience with cefiderocol compared to trials and international guidelines.

Results from the phase 3 RCT CREDIBLE comparing cefiderocol to the best available therapy in patients with CR-GNB showed an unexpected increase of mortality in the subset of patients with CRAB [10], especially in the setting of ventilator-associated pneumonia and BSI, but several limitations have to be acknowledged, such as the heterogeneity in the comparator arm and the relatively small number of patients. Also, there is a gap to investigate between results from the RCT CREDIBLE and the real-world observations, as demonstrated by recent study from the clinical practice [11], showing that patients with CRAB infections who received cefiderocol have a lower risk of 30-day mortality compared to controls under colistin-containing regimens.

Seven RCTs have investigated the role of combination therapy for CRAB infections and only 1 of the 7 trials indicated a potential benefit with combination therapy. Colistin vs. colistin + rifampin: 210 ICU patients with invasive CRAB infections, no difference in 30-day mortality. Higher microbiological cure in the combination arm [12]. Colistin vs. colistin + rifampin: 43 patients with CRAB pneumonia, no difference in hospital mortality [13]. Colistin vs. colistin + rifampin: 9 patients with colistin-resistant Acinetobacter infections, no difference in clinical outcome [14]. Colistin vs. colistin + fosfomycin: 94 patients with CRAB infections, no difference in 28-day mortality. Higher microbiological cure in the combination arm [15]. Colistin vs. colistin + meropenem: 312 patients with CRAB VAP, HAP bacteremia, no difference in 28-day mortality [16]. Colistin vs. colistin + meropenem: 328 patients with A- baumannii MDR BSI and pneumonia, no difference in 28-day mortality [17]. Colistin vs. colistin + HD ampi/sul (24 g/die): 39 patients with CRAB VAP, clinical improvement by day 5 in 16% vs. 70% (*p* < 0.01) [18].

We discussed above the issue of combining colistin with other drugs for the treatment of MDR *Acinetobacter baumannii*. In addition, we report 3 retrospective cohort studies of critically ill patients with CRAB infections performed at Chiang Mai University Hospital: colistin vs colistin + vancomycin with no significant differences in 30-day mortality, clinical response, microbiological response and the nephrotoxicity rates [19]; colistin vs colistin + meropenem with meropenem plus colistin caused a reduction in 30-day mortality, higher clinical and microbiological responses, and did not increase nephrotoxicity compared to colistin monotherapy. Furthermore, 30-day mortality was significantly related with age, receiving vasopressor, having malignancy, and the APACHE II score [20]; colistin + imipenem loading dose (LD) vs colistin + meropenem LD with the combination of LD colistin and meropenem provides a better survival rate for treating CRAB [21].

In this case a combination therapy with colistin and cefiderocol was preferred for this patient with severe deterioration of respiratory status. Despite the favourable minimum inhibitory concentration of *Acinetobacter* to colistin, the unpredictable pharmacokinetic/pharmacodynamic profile of this drug is a treatment limit. In fact, colistin has been associated with a low concentration in the epithelial lining fluid [22], which is particularly inconvenient when treating critically-ill patients. The addition of cefiderocol represented an effective treatment as rescue therapy in this severely-ill, immunocompromised patient with respiratory deterioration prior to VAP onset, displaying an interesting profile of intrapulmonary penetration [23].

Particularly, besides the favorable clinical outcome, it seemed to have led to microbiological cure: in fact, the molecular research of MDR-AB on tracheal aspirate was negative following combination therapy with colistin and cefiderocol in association with the source control of empyema with thoracic drainage. In addition to efficacy, the therapy was also safe and well tolerated.

Despite the severity of the clinical picture to which hospitalization in intensive care, VAP, empyema, BSI, HIV-related immunosuppression and concomitant COVID-19 have contributed, with the combined cefiderocol-colistin therapy, therapeutic and microbiological success were achieved.

Cefiderocol has demonstrated in vitro activity against *Acinetobacter baumannii* and is currently the only b-lactam displaying such activity against this microorganism. None of the novel b-lactamase inhibitors (avibactam, vaborbactam, and relebactam) have demonstrated activity against Acinetobacter oxacillinases, nor activity against metalloenzymes, and as a result, the new combinations do not display any activity against *Acinetobacter*.

Albeit limited, several real-life experiences reporting the use of cefiderocol as monotherapy (concerning the treatment of such infections) in the context of rescue therapy, but combo therapy with colistin doesn't investigate. In clinical centers where ampicillin/sulbactam and cefiderocol susceptibility testing is not available, in consideration of the epidemiology of circulating *Acinetobacter baumannii* strains, the sensitivity of the *A. baumannii* to colistin (shown on the susceptibility test) and the immunocompromised state of patient with respiratory deterioration, we can associate colistin (certainly active but with low intrapulmonary penetration) with cefiderocol (potentially active and with major profile of intrapulmonary penetration).

Our report suggests the potential role of cefiderocol in the treatment of CRAB infection even in HIV infected patients, adding new information on the clinical experience with this drug. Well-designed clinical studies are needed to generalize these findings, allowing for a more defined clinical use of cefiderocol, especially concerning infections by *Acinetobacter baumannii*.

## Declaration of Competing Interest

A.C. received support for travel to meetings from ViiV Healthcare, A.B. received speakers’ honoraria from ViiV Healthcare, and fees for attending advisory boards from Janssen-Cilag. S.D.G. was a paid consultant or member of advisory boards for Gilead Sciences, ViiV Healthcare, Janssen-Cilag, Merck Sharp & Dohme and Bristol-Myers Squibb. All other authors: none to declare.
